# Diptool—A Novel Numerical Tool for Membrane Interactions Analysis, Applying to Antimicrobial Detergents and Drug Delivery Aids

**DOI:** 10.3390/ma14216455

**Published:** 2021-10-27

**Authors:** Mateusz Rzycki, Sebastian Kraszewski, Marta Gładysiewicz-Kudrawiec

**Affiliations:** 1 Department of Experimental Physics, Faculty of Fundamental Problems of Technology, Wroclaw University of Science and Technology, 50-370 Wroclaw, Poland; marta.gladysiewicz-kudrawiec@pwr.edu.pl; 2 Department of Biomedical Engineering, Faculty of Fundamental Problems of Technology, Wroclaw University of Science and Technology, 50-370 Wroclaw, Poland; sebastian.kraszewski@pwr.edu.pl

**Keywords:** surfactants, numerical tool, drug delivery, free energy calculation, molecular dynamics, lipid membranes

## Abstract

The widespread problem of resistance development in bacteria has become a critical issue for modern medicine. To limit that phenomenon, many compounds have been extensively studied. Among them were derivatives of available drugs, but also alternative novel detergents such as Gemini surfactants. Over the last decade, they have been massively synthesized and studied to obtain the most effective antimicrobial agents, as well as the most selective aids for nanoparticles drug delivery. Various protocols and distinct bacterial strains used in Minimal Inhibitory Concentration experimental studies prevented performance benchmarking of different surfactant classes over these last years. Motivated by this limitation, we designed a theoretical methodology implemented in custom fast screening software to assess the surfactant activity on model lipid membranes. Experimentally based QSAR (quantitative structure-activity relationship) prediction delivered a set of parameters underlying the Diptool software engine for high-throughput agent-membrane interactions analysis. We validated our software by comparing score energy profiles with Gibbs free energy from the Adaptive Biasing Force approach on octenidine and chlorhexidine, popular antimicrobials. Results from Diptool can reflect the molecule behavior in the lipid membrane and correctly predict free energy of translocation much faster than classic molecular dynamics. This opens a new venue for searching novel classes of detergents with sharp biologic activity.

## 1. Introduction

**Figure 1:**
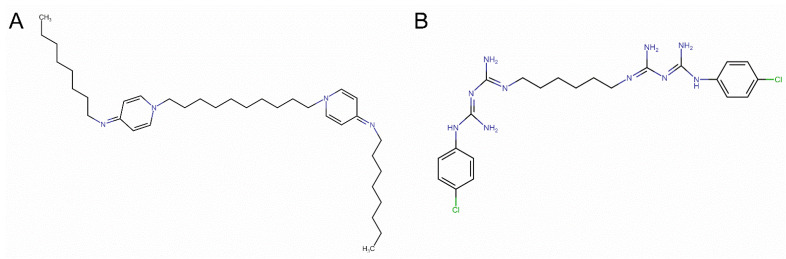
The chemical structures of antibacterial agents (**A**) OCT and (**B**) CHX.

The increasing problem of antibiotic resistance was identified by The World Health Organization as one of the current major threats to global health [1]. Unless novel medicines can be developed, millions more people may die each year. One of the approaches to limit this phenomenon involves the application of antibacterial candidates with a broad spectrum of activity. The molecular target of those compounds is not well defined in microbial cells, but it is safe to guess that it could be the cell membranes or their components. The complex interaction with various cellular structures may significantly reduce the bacteria’s resistance development [2,3]. Cationic Gemini surfactants belong to a class of compounds with broad-spectrum activity, effective against Gram-positives and Gram-negatives even in low concentrations, and are also used in drug nanoparticles delivery. Beyond biomedical applications, specific Gemini detergents are also used in the paint industry as corrosion inhibitors [4,5]. In the recent decade, only a few new classes of surface-active compounds have been discovered and have attracted the attention of researchers and industrial innovation units. Recently, several novel groups of bio-active detergents have been synthesized and reported [6,7,8,9]. The latest classes of amphiphilic-structured compounds are formed by Gemini surfactants, which are made of two aliphatic hydrocarbon chains and two hydrophilic head groups bonded by a rigid or flexible spacer. The spacer can be a symmetrized bonding that uses two of the same molecules (e.g., a disulfide bridge), whereas the head groups can be composed of: phosphate, sulfone, carboxyl, sulfate ethylammonium, or pyrrolidine residues. Gemini surfactants were already proven experimentally to have a high antimicrobial capacity [10,11,12], and in the future may be applied in facilitating drug delivery across membranes [13,14]. Unfortunately, direct antibacterial effectiveness comparison between groups of agents is often unfeasible due to the various bacterial strains used, and multiple, not always coherent, approaches of minimum inhibitory concentration (MIC) measurements. Thus, a delivery of clear, specific structural parameters of agents that may enhance antimicrobial action remains elusive. Many attempts were proposed to explain key structure components or the membrane disruption mechanism itself [15,16,17,18,19,20], without clear success. Instead of incoherent experimental procedures among different laboratories, a theoretical approach would better address this issue, offering additional insights at the molecular level. Molecular dynamics (MD) simulation can be successfully applied to explore the behavior and activity of synthesized molecules, opening the middle-throughput analyses in silico [21]. In MD the interactions are explored by solving Newton’s laws of motion for the system (i.e., bacterial lipid membrane and antimicrobial agent), fully taking into account the water environment. Those simulations may reflect the real biological environment and overcome the experiment shortcomings up to the atomic level. The antibacterial effect of surfactant activity has already been studied in this way many times, and the interactions of the model molecules, octenidine (OCT) and chlorhexidine (CHX) with mimetic membranes, have been repeatedly described [21,22]. The chemical structures of antimicrobial agents OCT and CHX are presented in Figure 1.

In general, the molecular dynamics approach is a very versatile method but requires a lot of resources and time to effectively generate the results. Hence, molecular docking tools dedicated to protein-drug interactions have emerged in the field of pharmaceutical research. The molecular docking approach, based on quickly scored interactions without an explicit water environment presence, becomes an increasingly important tool for discovering new drugs by opening the high-throughput analyses in silico [23,24]. It offers an opportunity to model at the atomic level the interaction of small molecules with proteins, however, explaining the fundamental biochemical processes. Although docking is a much more lightweight and way faster method than MD, it has some serious limitations, such as the impossibility of docking molecules to the entire lipid membrane structure due to its essential dynamics, which molecular docking does not address [23].

As there is an increasing interest to rapidly assess the molecular interactions of selected molecules with lipid membranes, we decided to provide a numerical tool in response to these needs. Here we present a novel and unique tool that covers the gap between MD and docking approaches, allowing the investigation of agent-lipid membrane interactions as fast as docking does but with MD precision enabling high-throughput analyses for interactions between detergents and lipids in antibacterial context. Due to the lack of unambiguous parameters determining the effectiveness of molecule-lipid membrane interactions, we used the quantitative structure-activity relationship (QSAR) method [25] to extract statistically significant properties for previously well-studied model Gemini detergents, OCT and CHX. In detail, employing data from experimentally verified various Gemini molecules delivered by Minbiole group [15,26,27,28,29,30], we selected 138 agents and derived a quantum QSAR, where only macroscopic descriptors such as dipole moment, partition coefficient (logP), and some structural parameters occurred as most significant for effective interactions with lipids. Descriptors were chosen to provide antimicrobial performance, comparable to standardized MIC form literature study, to ensure reproducibility of macroscopic biological phenomenon of membrane dissolution.

In this work, we introduce the methodology and the screening tool for a rapid determination of the Gemini agent affinity to various types of homogenous lipid membranes providing particle trajectory visualization and free energy analysis. We validated our tool on two commercially available and frequently studied antibacterial molecules: OCT and CHX. Additionally, we compare the results from our tool with the free energy calculations obtained directly from MD with the adaptive biasing force (ABF) approach for different lipid membranes.

## 2. Materials and Methods

### 2.1. Background

The QSAR method allows correlating the biological activity of the compound with valued physiochemical properties. Hydrophobic, electronic, or steric properties (descriptors) allow the analyzing of large databases of many agents that evoke a biological response in various molecular pathways. Some descriptors are difficult to clarify, taking into consideration the indirect representation of chemical structures. To counteract this, QSAR methods are powerfully supported by many mathematical approaches starting from multiple linear regression (MLR) [31,32,33] through an artificial neural network (ANN) [34,35,36,37] to machine learning (ML) [38,39]. These studies have a significant impact on recognition and understanding of the molecular mechanism of drug action, allowing the design of new and more specific candidates. Hence, since the last decade, chemoinformatics [40] has been booming and plays a significant role in discovering new drugs [41]. Moreover, the European Commission in the New Chemical Policy REACH (Registration, Evaluation and Authorization of Chemicals, European Union) recognized the method as relevant, whereas the information from alternative sources may assist in determining the presence of insecure properties of the substance, and may, in some cases, substitute for the results of animal tests [42]. In this study, our purpose was to create QSAR GA (genetic algorithm) on a set of 138 cationic Gemini surfactant molecules and to define parameters that significantly affect the potency of the antibacterial effect. In the QSAR regression model, the regression coefficient (*r*^2^) indicates the relationship correlation, whereas the cross-validation regression coefficient (*CVr*^2^) indicates the prediction accuracy of the model. Our major goal was to demonstrate that the phenomenon is not always straightforward, hence we focus on parameters to assess which are significant. We created several quantum QSAR linear regression models (quantum means with descriptors describing molecule at quantum level) based on delivered set of coherent MIC values for *Pseudomonas Aeruginosa, Escherichia Coli, Enterococcus Faecalis,* and *Staphylococcus Aureus* using SCIGRESS 3.3.3 (Fujitsu Ltd., Tokyo, Japan). Based on our dataset, 472 descriptors were found for every bacteria strain. We performed a 5-descriptor QSAR model for particular bacteria strains and subsequently, we decided to focus on the three most frequently occurring and most significant, but not bond-related parameters in the output– i.e., dipole moment, logP, and size, successively (see Table 1).

The 1.0/Csp^3^ bonded to 2C is an overlapping structural parameter associated with hybridized carbon atoms attached to exactly two carbon atoms; as it cannot be directly implemented into the calculation core as a force source, it was omitted. Based on these results, we employed dipole–dipole interaction as a calculation engine, thus implementing the first significant relationship from the obtained models. A more detailed description of motion is described in the Theory section. Partition coefficient (*logP*) and *length/width* (*size*) were included as subsequent parameters in our screening tool. The coefficient *logP* determines a measure of lipophilicity of a compound. i.e., drug and describes its ability to pass through the cell membrane barrier. It is identified as a drug distribution ratio between aqueous and organic layers at an equilibrium state [43]. To define the partition coefficient, arbitrary concentration units are often used (instead of potentials); however, the use of molarity is also useful, thus the logarithm of the partition coefficient can be determined from the chemical potentials [44].

(1) $$log_{10}P=\frac{\mu^{w}-\mu^{o}}{RT\;\ln\left(10\right)}=\frac{\Delta H_{\mathrm{transfer}}}{RT\;\ln\left(10\right)}$$

where *µ^w^* and *µ^o^* are excess chemical potentials of the Gemini agent in water and octanol respectively, R is the gas constant, *T* is the temperature, and 2.303 equals ≈ ln(10). In this approach, we decided to introduce the structural parameter (*size*) which defines the *length/width* of the molecule. For general use, the molecular structure is considered as a sphere, described by radius *R*, thus the degrees of freedom are reduced.

The particular lipid and Gemini agent dipole moments were extracted from molecular dynamics trajectories using MOPAC 2016 (Molecular Orbital PACkage, Stewart Computational Chemistry, Colorado Springs, CO, USA) software with PM7 method [45,46]. Briefly, the specific approximation for intermolecular interactions used in PM7 was parameterized using experimental and high-level ab initio reference data, augmented by a new type of reference data intended to better define the structure of parameter space [46]. Dipole moments were derived for each lipid and agent in the system from 200 ns trajectories, and the mean values and standard deviations are used as inputs to our screening tool.

### 2.2. Theory and Calculation

In our approach, we accumulate particle motion by reducing this motion to that of an agent particle in a conservative field. The agents in this case are the OCT and CHX particles. The cell membrane was treated as a system of dipoles *µ* placed in a medium described as a dielectric permittivity *ε_r_.* The molecule enters the membrane from the aqueous environment described by the parameters *ε_r_* and viscosity η. The particle is treated as a dipole attached to the sphere and moving in conservative field derived from dipoles constituting the cell membrane. Membrane dipoles are generated from the normal distribution of range of values defined by the mean lipid dipole value +/− standard deviation, which were formerly calculated for each lipid type from the 200 ns of molecular dynamics trajectory (see Table 2). As a result, a unique membrane is generated automatically by our Diptool engine and takes part in the further computation procedure. In this conservative field we can describe force *F* corresponding to the potential energy *E_p_*:

(2) $$\vec{F}=-\frac{dE_{p}}{d\vec{r}}$$

Energy *E_p_* comes from each lipid constituting membrane at distance *r*, and generates dipole-dipole interaction, which can be calculated considering the electrostatic interaction between the membrane-forming charges and the dipole of the studied molecule:

(3) $$E_{p}=\sum \frac{\mu_{i}\cdot \mu }{4\pi \varepsilon_{0}\varepsilon_{r}r_{i}^{3}}$$

where *µ_i_* is dipole element of membrane and *ε_r_* is relative dielectric permittivity that equals 88.0 for water and 4.0 [47,48] for the membrane, and *r_i_* is the distance between two dipoles. The summation proceeds over all dipoles in the considered membrane. Hence, only the electrostatic interaction between two dipoles is considered, omitting the rotation of dipoles relative to each other. However, it is a good enough approximation in a situation when one treats the particle as a point mass. Such a procedure can be used if the size of the molecule is much smaller than the size of the membrane, which is the case. Velocity and acceleration vectors can be described by:

(4) $$\vec{v}=\vec{a}\cdot \Delta t$$

(5) $$\vec{a}=\frac{\vec{dv}}{dt}=-\frac{1}{m}\frac{dE_{p}}{\vec{dr}}$$

where *m* is the particle masses and in our case equal 623.84 g/moL and 505.452 g/moL for OCT and CHX, respectively. Position vectors of particles are described by coordinates *r* = (*x*, *y*, *z*). Numerical formulas give the equations corresponding to Verlet algorithm [49]:

(6) $$\vec{r^{n}}=\vec{r^{n-2}}+2\Delta t\vec{v^{n}}$$

(7) $$\vec{v^{n+1}}=\vec{v^{n-1}}-2\Delta t\frac{1}{m}\frac{d}{d\vec{r}}E_{p}\left(r^{n},t^{n}\right)$$

where ***v***= (*v_x_*, *v_y_*, *v_z_*) is a velocity vector, and *n* numerate step of calculation. The potential energy *E_p_* of the dipole–dipole interaction is a function of the particle position and time *t*. The iteration producing the velocity is carried forward to the iterated position. Such a procedure affects the stability of the algorithm, thus we added the term of resistance force *F_R_* corresponding to the environment viscosity η:

(8) $$\vec{F_{R}}=-b\vec{v}=-6\pi \eta R\vec{v}$$

where *R* is a sphere radius of an agent particle. In our calculation we put *R* = 10^−14^ m and η = 0.89 × 10^−3^ Pa·s for water and 934 × 10^−3^ Pa·s for the membrane. We assume the particle is an ideal sphere with a radius of *R*, which is related to *size* parameter. In this way, the number of degrees of freedom was limited. The radius is so small that the sphere can be treated as a point mass. Integrating resistance force to the propagation equations we get for position and velocity:

(9) $$\begin{array}{c} \vec{r^{n}}=\vec{r^{n-2}}+2\Delta t\vec{v^{n-1}}\\ \vec{v^{n+1}}=\vec{v^{n-1}}-2\Delta t\frac{1}{m}\frac{d}{d\vec{r}}E_{p}\left(r^{n},t^{n}\right)-\frac{b}{m}\vec{v^{n-1}}2\Delta t\; \end{array}$$

Gibbs free energy can be determined deriving acceptance rules for NPT ensemble (where *N* is number of particles, *P* is pressure, and *T* is temperature), as it is also in MD. To define the probability density, and knowing the conjugate variables (*N*→μ, *V*→P, *T*→*E*) in the NPT ensemble μ, *V*, and *E* will vary, thus in given probability distribution, moves are accepted to satisfy a detailed balance. Assuming that in the thermodynamic limit, all ensembles are equivalent, the change of free energy can be calculated as the work between the states *A* and *B* [50,51]:

(10) $$\Delta G=W_{A\to B}\;$$

The system can be described using Hamiltonian *H*(*v_x_*, *v_y_*, *v_z_*, *x*, *y*, *z*). If the temperature and volume of the system is maintained to calculate the change in Gibbs free energy, it is sufficient to determine this quantity. This Hamiltonian consists of the potential energy *E_p_* and the kinetic part considering the velocity:

(11) $$H\left(v_{x},\;v_{y},y_{z},x,y,z\right)=E_{p}+\frac{mv^{2}}{2}$$

The kinetic energy of our molecule is limited to *k_b_T* (where *k_b_* is the Boltzmann constant), avoiding excessive velocity. Such a procedure allows eliminating the unrealistic results. Once the Hamiltonian in each point has been determined, one can calculate the change in Gibbs free energy between the initial state *A* and the final state *B*:

(12) $$\Delta G_{A\to B}=H_{B}-H_{A}$$

If non-conservative forces are acting in the simulated system, we must also consider the kinetic energy component. Above the *k_b_T* constraint, the kinetic energy is only affected by the energy resulting from the dipole–dipole interaction, so the Hamiltonian then simplifies. In addition, we have considered the situation that during the agent’s transfer the environment changes from aqueous to membrane (*logP* parameter). Then, when a particle approaches the membrane, it must overcome additional potential (Equation (1)), and the Hamiltonian takes form:

(13) $$\Delta H_{\mathrm{transfer}}=\ln\left(10\right)RTlog_{10}P$$

By consequence, if in the next step of iteration there is a change in *ε_r_* then the Gibbs energy is calculated according to:

(14) $$\Delta G_{A\to B}=H_{B}-H_{A}+\Delta H_{\mathrm{transfer}}$$

This approach also works when the particle is pushed out of the membrane.

The presented numerical algorithm allows us to determine the trajectory and Gibbs free energy for any particle represented as a dipole in a conservative field produced by other dipoles. This algorithm is very simplified and assumes only interactions between dipoles, with minor corrections (η and *logP*), and omits other interactions. However, qualitatively it allows us to estimate the macroscopic response of the membrane system with fair accuracy.

### 2.3. Molecular Dynamics Validation

The proposed methodology was verified using free energy profiles in molecular dynamics simulations. The all-atom models of the membranes were generated using CHARMM-GUI membrane builder [52]. In this work PC (1-palmitoyl-2-oleoyl-glycero-3-phosphocholine) and PG (1-palmitoyl-2-oleoyl-sn-glycero-3-phospho-(1′-rac-glycerol)) lipids were selected for the comparison of the agents’ behavior in neutral (100% POPC) and negatively charged (100% POPG) environments, reflecting mammalian and bacterial inner membranes, respectively. The TIP3P water model was employed and counterions were included in PG. Finally, both membranes were composed of 200 lipids (100 per monolayer).

MD simulations were performed using the NAMD (version 2.14, University of Illinois, Urbana, IL, USA) package [53] with the CHARMM36 force field [54]. Calculations were carried out in the NPT ensemble (constant number of particles, pressure, and temperature) at constant pressure (1 atm) and temperature (300 K) using the Langevin piston method and Langevin dynamics [55]. Short and long-range interactions were computed every 1 and 2 time-steps, respectively. Long-range electrostatic forces were evaluated using the particle mesh Ewald (PME) method [56], which allowed us to employ the integration timestep of 2 fs. Finally, 200 ns of pure membrane trajectories were produced and taken as an input to the free energy method.

**Figure 2:**
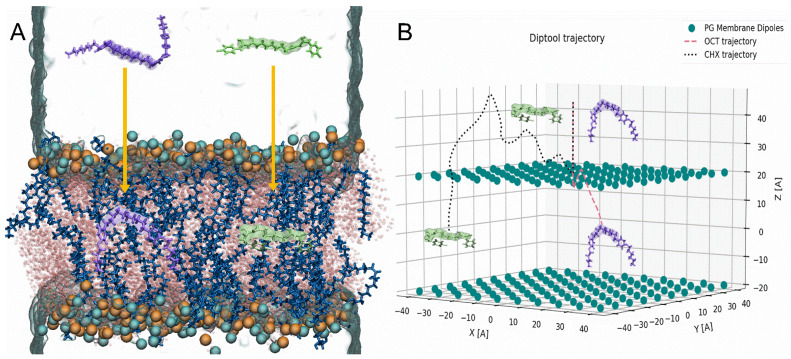
Initial and final trajectories obtained from (**A**) ABF−method, (**B**) Diptool software on PG membranes. CHX: green sticks and surface, OCT: violet sticks and surface, POPG: blue and pink sticks, phosphorous atoms: cyan beads, nitrogen atoms: orange beads and PG membrane dipoles: teal beads.

From a bunch of available protocols, the ABF method was selected because it is an established and precise approach [57,58]. The free energy profiles were obtained using the ABF extension implemented in NAMD software. The integrated collective variables module [59] was applied to the delivered MD simulation protocol. The agent molecules were placed 45 Å over the bilayer center, and the reaction coordinate is consistent with the membrane normal vector between the center of mass of the membrane and the center of mass of the agent spacer. The number of atoms in antimicrobial particles was intentionally reduced to limit the degrees of freedom and molecules’ fluctuation. Therefore, the most rigid region (spacer) of OCT and CHX was selected using C14–C23 atoms and C7–C16, respectively (highlighted in Figure 2). The minimal sampling of 50,000 samples along for each step was applied, employing 0.2 Å width step along the reaction coordinate. Finally, at least 0.7 µs was produced to obtain each free-energy profile. For visualization and analysis purposes, Visual Molecular Dynamics was used (VMD) [60]. Here OCT and CHX molecules were employed from our previous work where the description of molecular parametrization was presented in detail [21].

### 2.4. Data Visualization and Analysis

Diptool is provided with a dedicated engine written in C++ and visualization package implemented in Python (Python Software Foundation, Wilmington, DA, USA.) and requires version 3.7 or higher. Three-dimensional trajectories and energy profile plots are generated based on delivered input from the Diptool engine and MD ABF. It uses the following libraries: matplotlib, math, pyplot, numpy, seaborn. The Diptool engine is compilated C++ code (*Diptool_engine.exe*) where initial parameters such as dipole moments and errors, membrane size, area per lipid (APL), Gemini agent mass, and its dipoles need to be delivered in a parameter text file (*param.txt*). Diptool should be executed from an included python script (Diptool_run.py), in which the tool engine is embedded and automatically launched, and after finished calculations a trajectory and energy plots are generated. In the meantime, three files with plotted membrane position, agent trajectory, and energy profiles are produced, respectively. In *membrane.txt* file the dipoles arrangement in X, Y, Z direction are stored, *data.txt* include the agent trajectory in the X, Y, Z axes, and finally *energy.txt* contain the energy profile with respect to the bilayer normal—Z direction. The python visualization code was commented including several hints to simplify the usage. Apart from Diptool engine calculations, the MD free energy profiles may be visualized for quick comparison. In the Supplementary materials, complete Diptool software with exemplary files for initial runs is delivered.

Significance tests and plots were performed using OriginLab Origin 2018 software (OriginLab, Northampton, MA, USA). Specifically, one-way ANOVA was applied with post-hoc Tukey test to determining significance between individual populations with the significance level at 0.05.

## 3. Results and Discussion

The membranes have been described in Diptool as a set of dipoles that interact with each other and with surfactant dipoles as well. To obtain dipole moments of particular lipids and agents, corresponding trajectories from MD were employed. From the 200 ns of simulations we extracted dipole moments for every molecule in the bilayer, resulting in set of 400,000 dipoles in the X Y Z direction. The average and the standard deviation of the Diptool input is presented in Table 2. The lipid positions and mobility affect the wide range of obtained data, which results in the high fluctuations of standard deviation. In this approach, we decided to avoid averaging the error to derive a wider range for random membrane components generator and deliver a complex expertise of membrane-agent interactions. Such a procedure allows employing many types of lipids reflecting many membrane species supposing that dipole moments are known. Additionally, at this stage a membrane size may be adjusted in all axes, whereas a number of lipids is automatically calculated based on the given volume and APL. In this work we decided to compare the Gemini agents behavior in neutral and negatively charged environments using PC and PG lipids [61,62]. These are commonly used in both experimental and theoretical work, because the latter is one of the major components in bacterial bilayer structures, whereas PC is mimicking eukaryotic cell membranes. That system configuration often allows demonstrating distinct and selective behavior of active compounds such as OCT and CHX in various membrane systems [21,63,64]. A different behavior may also be visible due to those particular lipids yielding various dipole moments, especially in the Z-axis and in the total value as well.

Similarly, as in MD, Diptool allows to register, i.e., trajectories and energies and analyze them afterward. However, employing the average and standard deviation of system components in the tool may deliver slightly different results whenever used. That combination allows for a more comprehensive case study about selected surfactants accompanied by given lipids. In Figure 2 the snapshots from MD trajectories (Figure 2A) and Diptool (Figure 2B) are presented where OCT and CHX behavior were investigated in membrane systems. In all cases, an agent particle was placed 45 Å above the membrane center, and their trajectories seem to present similar features. The surfactant molecule approaches the membrane surface, penetrating it and facing the bilayer center afterward. Although Diptool uses a different core of motion, the modeled trajectories correspond to those from both steered and classical MD. The Diptool trajectories may vary significantly in the subsequent runs because dipole location and value are different. However, the starting and ending point reached by tested molecules remain consistent. Based on given trajectories, significant differences in behavior between CHX and OCT may be noticed. In the case of OCT, a small molecule fluctuation at the membrane interface may be observed, whereas CHX particles indicated higher resistance and took a little longer to reach the membrane center (see Figure 2B).

**Figure 3:**
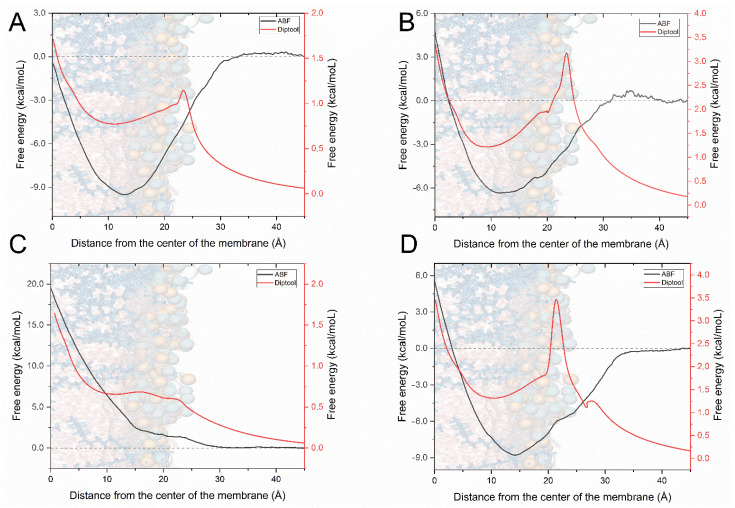
The comparison between MD−ABF and Diptool free energy calculation, marked in black and red, respectively. (**A**) Free energy transfer for OCT in PC membrane, (**B**) Free energy transfer for OCT in PG membrane, (**C**) Free energy transfer for CHX in PC membrane, (**D**) Free energy transfer for CHX in PG membrane.

The second main feature derived from Diptool is the system energy calculation, which corresponds to the free energy calculation provided by the MD−ABF method. The results from both methods for OCT and CHX molecules are presented in Figure 3. An essential part of the free energy determination is the difference associated with the molecule translocation toward the membrane from bulk water to bilayer center—ΔG_trans_ [57]. The free energy calculations illustrated that in a neutral membrane, OCT spontaneously crosses the bilayer interface, exhibiting the deepest well of ~9 kcal/mol; hence, it is the most thermodynamically favorable location (see Figure 3A). This corresponds to our previous studies, where we indicated theoretically and experimentally that OCT locate in the carbonyl−glycerol region preferentially [21]. Subsequently, an expected high energy barrier towards the membrane center occurs as also reported in other work focused on the transport of charged particles from water to the hydrophobic core [57,65]. Although surfactant can easily access the membrane, the translocation to another leaflet is energetically demanding. As stated, the ΔG_trans_ is the difference between ΔG_bulk_ and ΔG_core_ [57] and yields ~0.5 kcal/mol. The core motion of Diptool is unique, therefore, the final energy plot also indicates individual regions. First, a small energy barrier of ~1 kcal/mol may be noticed at the membrane interface, which is related to dipole−dipole interactions between particle and the zwitterionic lipids. Because the agent trajectory is correlated with membrane normal, and a corresponding vector dipole moment does not yield extreme values, only a small peak representing the barrier is visible. Similarly, as in the ABF−method, the local minimum in the membrane is reached near the carbonyl−glycerol area. Moving forward, facing the bilayer center, the energy barrier occurs again from the hydrophobic core. The total OCT’s ΔG_D.trans_ using Diptool reached ~1.7 kcal/mol, which successfully screened the agent behavior compared to the extensive ABF calculations. In the case of charged PG membranes (see Figure 3B), the free energy associated with OCT indicates slight fluctuations near the membrane surface of ~0.5 kcal/mol, while subsequently a ~6 kcal/mol well is reached. The largest barrier to overcome was faced in the direction of the membrane center yielding total a ΔG_trans_ of ~4.5 kcal/mol. In the results from Diptool, the system differences may be clearly visible, due to large peak of ~3 kcal/mol at the membrane entry caused by negatively charged lipids. Further, local membrane minimum is reached (~1.3 kcal/mol) and the final hydrophobic barrier occurred as in previous cases, ending with a total ΔG_D.trans_ of ~3.3 kcal/mol. It should be noted that local minima in PG membranes are shifted toward the bilayer center relative to the PC ones, which indicates that OCT prefer to stay a bit deeper in the negatively charged membranes.

Another, much different energy profile associated with CHX behavior was reported in PC membranes (see Figure 3C). Interestingly, the molecule does not access the membrane spontaneously as OCT does. Analogous limited agent–membrane interaction was observed in our previous report [21] as several CHX molecules incorporated into membrane; however, some of them stayed in the bulk water. Here, a small barrier of ~1.9 kcal/mol accompanies molecule entry to membrane, and further at carbonyl−glycerol region strikes up to ~20 kcal/mol. This specific behavior may be related with low logP coefficient logP = 5.48, whereas for OCT logP = 9.25. This may indicate that the substance has a high tendency to locate in the outer part of the membrane. This is in strong agreement with the results from Diptool, as a low energy barrier is at the membrane interface, with a local minimum in the membrane at hydrophilic heads. The further peak corresponds to the one observed in the ABF method; however, registered values vary a lot, as ΔG_trans_ and ΔG_D.trans_ equal ~20 and ~1.6 kcal/mol, respectively. In the case of a negatively charged PG bilayer, the agent demonstrated a distinct energy profile (see Figure 3D). A negatively charged membrane is ideal for CHX, which find the sweet spot at local minima of ~9 kcal/mol. Similar to the other cases, the biggest energy barrier occurs when facing bilayer center, ending with a total ΔG_trans_ of ~5.8 kcal/mol. Diptool results indicate a big energy barrier from negatively charged lipids at 22 Å from the bilayer center, while afterward local minimum is settled below the carbonyl−glycerol region. Here the final ΔG_D.trans_ equals ~3.5 kcal/mol, which fairly reflects the accurate energy calculations from ABF−MD. Higher Diptool energy may indicate more effective antimicrobial action. It should be noted that proposed energy analysis is one of the means of evaluating membrane−agent interactions. Because Diptool is a screening tool, extensive calculations are needed for delivering a comprehensive outcome, especially in drug-delivery studies. Given results may significantly accelerate and narrow down the group of tested compounds; however, experimental or theoretical confirmations should be delivered additionally.

To this end, Diptool is a screening tool whose results correspond with those derived from free energy calculations in MD. Although it is based on a modified Verlet algorithm, its capabilities and accuracy are limited and not so precise compared to a classical MD approach. The greatest advantage of the tool is its performance. Used algorithm and modifications combined in the C++ engine makes it reliable, fast, and lightweight software. The local performance test was conducted using AMD Ryzen 5 2600 CPU (AMD Santa Clara, CA, USA) supported with 16GB DDR4 RAM. Out of 100 runs, the average computation time lasted 5 ± 1 min, whereas to provide accurate and precise calculations from the ABF method, 64,800 ± 3527 min were needed using a 96 Intel Xeon E5 v3 CPUs (Intel, Santa Clara, CA, USA) together on four nodes, for the single system. Estimated acceleration for one molecule screening is thus about a million times comparing to steered MD study. That gives an enormous advantage to Diptool for screening a large database to select several candidates and deliver rigorous results afterward.

## 4. Conclusions

In this work, we presented a novel, self−made methodology supported with a software solution named Diptool—a screening tool for a rapid determination of the Gemini agent affinity to various types of homogenous lipid membranes delivering particle trajectory visualization and free energy analysis. In the presented study we introduced from scratch the genesis and background of delivered methodology, discussed the calculation core of the Diptool software, and finally validated and tested our tool with known antimicrobial candidates: OCT and CHX. Our results indicate that Diptool is able to generate accurate free energy profiles for Gemini surfactants significantly faster than other well-known, established, and more advanced methods. We used molecular dynamics studies to verify our assumptions highlighting the estimation of the free energy perturbation. Our calculations were provided on both neutral-PC and negatively charged-PG lipids, as the latter are major components in bacterial inner bilayers. We compared computations from Diptool and the ABF method from molecular dynamics focusing on the membrane−agent interactions presenting surfactant trajectory and free energy profiles. Despite much different theories of calculation, we indicated similarities in trajectories of OCT and CHX between the tested tools. In both cases, the agents were suspended in the bulk water and afterward penetrated the membrane toward the bilayer center, which allowed us to construct the free energy profiles. Here the final results of ΔG_D.trans_ and ΔG_trans_ indicate whether a given particle prefers to stay in the water phase, anchor in hydrophilic heads, or interact with hydrophobic core, as well as the energetic cost of that displacement. Delivered results indicate that the agent behavior in various lipid environments is well reflected with mostly corresponding translocation free energies, and hence expected macroscopic biological behavior. Additionally, our results confirm that a reduction in the membrane dipole results in reduced permeability of polar compounds [57,66]. We also provided a performance test that clearly indicates that Diptool is significantly faster than classical methods, reaching a one million-fold compared to the MD approach. We would like to clearly emphasize that provided software should be considered as a screening tool for rapid determination of agent effectiveness, enabling high−throughput screening for at least antimicrobial and drug−delivery aids based on detergent molecules. In this paper, we present the first version of Diptool; however, further development is planned. In the following versions we would like to deliver various lipid mixtures implementation, apply various solvents containing ion solutions, implement additional empirical parameters in the energy calculations to better follow the particle energy fluctuations, and offer graphical user interface (GUI) for more comprehensive, rapid, and accurate solutions for surfactant analyses.

## Figures and Tables

**Table 1:** Results from 5-Descriptor QSAR Calculations from *E. faecalis*, *E. coli*, *S. aureus*, and *P. aeruginosa* datasets with the Parameters’ Relative Importance. Frequently Appearing Descriptors were Bolded.

***E. faecalis***		***E. coli***	
**Descriptor**	**Relative Importance**	**Descriptor**	**Relative Importance**
***length/width***	**0.1920**	***length/width***	**0.2316**
***logP***	**0.8700**	***logP***	**0.8331**
***hydrophobic dipole moment***	**0.3655**	***hydrophobic dipole moment***	**0.3816**
*Hydrogen count*	−0.1418	*double bond count*	−0.2713
*1.0/Csp^3^ bonded to 2 C*	1.0000	*1.0/Csp^3^ bonded to 2 C*	1.0000
***S. aureus***		***P. aeruginosa***	
**Descriptor**	**Relative Importance**	**Descriptor**	**Relative Importance**
***length/width***	**0.3422**	***length/width***	**0.3644**
***logP***	**0.8358**	***logP***	**0.8829**
***hydrophobic dipole moment***	**0.4667**	***hydrophobic dipole moment***	**0.4782**
*atomic charge weighted positive area-atomic charge weighted negative area*	0.1409	*charge weighted nonpolar area*	−0.2206
*1.0/Csp^3^ bonded to 2 C*	1.0000	*1.0/Csp^3^ bonded to 2 C*	1.0000

**Table 2:** Derived Dipole Moments of Lipids and Agents from MD Simulations.

Dipole Moment in Particular Axis		X (D)	Y (D)	Z (D)	TOTAL (D)
Particle type	PC	0.34 ± 11.29	−0.37 ± 11.39	1.65 ± 8.44	18.27 ± 2.32
	PG	0.14 ± 10.17	0.29 ± 10.09	−35.08 ± 8.29	39.19 ± 4.69
	OCT	0.69 ± 7.08	2.12 ± 8.14	−0.51 ± 12.78	16.12 ± 4.55
	CHX	2.74 ± 15.46	2.21 ± 10.37	−0.55 ± 10.25	24.73 ± 8.72

## Data Availability

Most of the data are available in the main manuscript or generated by code attached to supplementary materials.

## Supplementary Materials

The following files are available online at https://www.mdpi.com/article/10.3390/ma14216455/s1, Software base files: Diptool_engine.exe, Diptool_run.py, param.txt; exemplary files, membrane.txt, data.txt, energy.txt.
